# The exosomal integrin α5β1/AEP complex derived from epithelial ovarian cancer cells promotes peritoneal metastasis through regulating mesothelial cell proliferation and migration

**DOI:** 10.1007/s13402-019-00486-4

**Published:** 2020-02-21

**Authors:** Xiaoduan Li, Meiling Tang, Qinyi Zhu, Xinjing Wang, Yingying Lin, Xipeng Wang

**Affiliations:** 1 Department of Gynecology, Shanghai First Maternity and Infant Hospital, Tongji University School of Medicine, Shanghai, 201204 China; 2 Department of Obstetrics and Gynecology, XinHua Hospital Affiliated to Shanghai JiaoTong University School of Medicine, Shanghai, 200092 China; 3 Department of Neurosurgery, RenJi Hospital, Shanghai JiaoTong University School of Medicine, Shanghai, 200127 China

**Keywords:** Epithelial ovarian cancer, Peritoneal metastasis, Integrin, AEP, HPMC

## Abstract

**Purpose:**

Epithelial ovarian cancer (EOC) is one of the most malignant cancers in the gynecologic system. Many patients are diagnosed at an advanced stage with disseminated intra-peritoneal metastases. EOC spreads via both direct extension and trans-coelomic spread. However, the interplay between human peritoneal mesothelial cells (HPMCs) and EOC cells is still ambiguous. We hypothesize that integrins (ITG) in HPMCs may play important roles in EOC metastasis.

**Methods:**

The expression of different integrin subtypes from HPMCs was assessed using Western blotting. The expression of integrin α5β1 (ITGA5B1) and its co-localization with asparaginyl endopeptidase (AEP) in HPMCs derived from EOC patients (EOC-HPMCs) were assessed using immunofluorescence. The role and mechanism of the exosomal ITGA5B1/AEP complex in HPMCs was assessed using both in vitro and in vivo assays. A retrospective study involving 234 cases was carried out to assess ITGA5B1 and AEP levels in circulating sera and ascites of EOC patients, as well as associations between ITGA5B1/AEP expression and overall survival.

**Results:**

We found that ITGA5B1was highly expressed and co-localized with AEP in EOC cells, and that the exosomal ITGA5B1/AEP complex secreted by EOC cells played an important role in the proliferation and migration of HPMCs. High levels of exosomal ITGA5B1/AEP were also found in circulating sera and ascites of EOC patients, and the expression of ITGA5B1/AEP in EOC tissues was found to be negatively associated with overall survival.

**Conclusions:**

Our data indicate that EOCs may regulate the function of HPMCs through exosomal ITGA5B1/AEP, which may be crucial for peritoneal metastasis.

**Electronic supplementary material:**

The online version of this article (10.1007/s13402-019-00486-4) contains supplementary material, which is available to authorized users.

## Introduction

Epithelial ovarian cancer (EOC) is one of the most malignant cancers occurring in the gynecologic system. In the United States alone, each year > 22,000 new patients are diagnosed with EOC and > 14,000 patients die from it [1]. Unfortunately, it is difficult to diagnose EOC in its early stages because of a lack of comprehensive laboratory tests and specific symptoms [2, 3]. Therefore, more than half of the EOC patients are diagnosed at advanced stages with widespread metastases. Also, due to high recurrence rates, the overall five-year survival is still low [4]. The key components of EOC progression are peritoneal dissemination, multiple organ metastases, refractory ascites and retroperitoneal lymph node involvement [5, 6]. The diagnosis of EOC recurrence and metastasis mainly relies on detection of the biomarker carbohydrate antigen 125 (CA125) [7] combined with medical imaging clues, which are limited in specificity and sensitivity. Thus, new biomarkers are urgently needed for early EOC and metastasis detection.

The conventional therapy of EOC at early stages is surgical excision, while cytoreduction followed by platinum- and taxane-based chemotherapy is used for EOCs at advanced stages [8, 9]. The mortality of EOC is directly associated with the prevalence of metastases. Approximately one-third of the EOC patients present with malignant ascites, and the presence of massive ascites is associated with a poor prognosis [10]. Trans-coelomic metastasis through the mesothelium correlates with ascites production. At advanced stages, small clusters of EOC cells in the ascites detach from the primary site and adhere to the abdominal peritoneum or omentum. When EOC cells adhere to the mesothelium of the peritoneal cavity, they proliferate, migrate and finally invade into the surrounding tissues. This indicates that EOC progression and dissemination may be regulated through cell-cell and/or cell-matrix associated adhesion mechanisms [11]. Thus, it is considered relevant to further explore contacts between EOC cells and HPMCs to unravel the mechanisms underlying peritoneal metastasis. It has been reported that inflammatory mediators and low pH can induce HPMCs to lose certain epithelial characteristics and to progressively acquire a fibroblast-like phenotype [12]. This epithelial to mesenchymal transition (EMT) process has also been shown to play an important role in tumorigenesis [13]. It is therefore relevant to explore the changes that take place in HPMCs and whether these changes are conducive to peritoneal EOC metastasis.

Integrins (ITGs), which are expressed on the surface of EOC cells, play important roles in the attachment of these cells to the mesothelial extracellular matrix (ECM) [14]. Integrins mediate cell-matrix and cell-cell interactions and constitute a family of transmembrane cellular receptors. They represent heterodimeric glycoproteins that consist of a larger subunit (α subunit, 120–170 kD) and a smaller subunit (β subunit, 90–100 kD) [15]. Integrins can transduce signals to regulate cell growth, proliferation, differentiation and adhesion, and they are expressed on various cell types including fibroblasts, endothelial cells and immune cells [16]. Some integrins are specifically expressed on EOC cells, such as α5β1, α2β1 and αvβ3 [16–18]. Several of them have been found to act as important mediators of EOC metastasis to the mesothelium of the peritoneal cavity. Integrin α5 (ITGA5) binds to integrin β1 (ITGB1). ITGA5B1 recognizes the arginine-glycine-aspartic sequence (RGD) on fibronectin, which is one of the most abundant proteins in the ECM of the EOC peritoneum and omentum [19]. As mentioned above, EOC metastasis starts with the attachment of cancer cells to mesothelial cells. This attachment can be inhibited by antibodies directed against ITGA5, ITGB1 or RGD peptides, which indicates that ITGA5B1 may play a crucial role in EOC cell binding to mesothelial cells [20]. Here, we assessed the expression of integrins from EOC-derived exosomes. Although AEP is a member of the C13 cysteine protease family, it generally presents as an inactive zymogen. Activation of AEP requires the removal of C-terminal and N-terminal pro-peptidase from pro-AEP [21]. AEP resides in acidic lysosomes or endosomes, takes part in intracellular protein degradation and plays an important role in atherogenesis [22], immunity [23] and cancer metastasis. AEP promotes cancer metastasis via degradation of the extracellular matrix [24]. It has been reported that high AEP expression correlates with a poor prognosis and a short survival time in prostate cancer [25], colorectal cancer [26], breast cancer [24], leukemia [27] and ovarian cancer [28].

Exosomes are small extracellular vesicles ranging from 30 to 100 nm in size that contain functional biomolecules such as DNA, RNA, lipids and proteins. Exosomes are key mediators of cell-cell communication, and the biomolecules contained within exosomes can be transferred to recipient cells [29]. As such, they provide crosstalk between tumor cells and the tumor microenvironment (TME), which includes endothelial cells, mesothelial cells, stromal cells, fibroblasts, extracellular matrices and infiltrating immune cells, through paracrine mechanisms [30]. Brain astrocyte-derived exosomes can, for example, promote brain metastatic cancer cell outgrowth by transferring miR-19a [31]. Exosomes may also contain cell surface-anchored proteases and carriers for adhesion molecules. It has been shown that exosomes exist in the ascites of ovarian cancer patients [32]. Since advanced EOC is always accompanied by malignant ascites formation, these studies suggest that exosomes may play an important role in EOC peritoneal metastasis [33].

Since the relationship between ITGA5B1 and AEP expression in EOC has so far rarely been reported, we aimed to explore the function of the ITGA5B1/AEP complex in EOC invasion. We found that HPMC regulation through the EOC exosomal ITGA5B1/AEP complex may be a crucial step in EOC peritoneal metastasis. Furthermore, we found that the ITGA5B1/AEP complex may serve as a new biomarker and/or therapeutic target for EOC patients.

## Materials and methods

### Patients and specimens

Epithelial ovarian cancer tissues and human omentums were collected from EOC patients and benign ovarian tumor volunteers at Shanghai First Maternity and Infant Hospital, Tongji University School of Medicine. A total of 234 surgical tissue samples were collected. None of the cancer patients received any chemotherapy before sampling. The histopathological diagnosis, stage and grade of the EOC samples were assigned according to the FIGO (International Federation of Gynecology and Obstetrics) classification. The objectives and implications of the results were explained clearly, and institutionally approved written informed consent was obtained from each participant. The study protocol was reviewed and approved by the Institutional Review Board of Shanghai First Maternity and Infant Hospital, in accordance with the Declaration of Helsinki.

### Enzymatic disaggregation of omentums for HPMC isolation

Omentums (approximately 5 cm^2^) were washed and incubated in 15–20 ml of a solution containing 0.125% trypsin and 0.01% EDTA (Trypsin-EDTA Solution, Sigma, 59428C) for 25 min at 37 °C with continuous rotation. Next, the suspensions were centrifuged at 1500 rpm for 10 min. The resulting cell pellets were washed once in low-glucose DMEM (HyClone, SH30021.01) containing 10% fetal bovine serum (FBS, Gibco, 10,099,141), re-suspended in low-glucose DMEM containing 20% FBS to a volume of 5 ml and seeded in 25 cm^2^ matrix-coated tissue culture flasks. Half of the medium was exchanged 24 h after seeding and, thereafter, fully replaced once every third day [34].

### Cell culture and transfection

Human EOC-derived SKOV3 cells were cultured in RPMI-1640 medium (HyClone, SH30809.01B) supplemented with 10% FBS and 1% antibiotics in a humidified atmosphere with 5% CO_2_ at 37 °C. SKOV3 cells were transfected with a luciferase reporter vector containing a puromycin resistance gene (SKOV3-luc) and grown in complete medium supplemented with 3 μg/ml puromycin (Shanghai MaoKang Biotechnology, MS0011-25MG). The cells were maintained in culture for no more than 10 passages and they were authenticated using short tandem repeat (STR) analysis every six months. The last test was conducted in February 2019. The cells were also authenticated by morphology, phenotype and growth characteristics, and routinely screened for mycoplasma contamination (Vazyme, MycoBlue™ Mycoplasma Detector, D101–02) at least every six months. The last test was performed in February 2019.

For stable transfection of the cells, ITGA5, ITGB1 and AEP lentiviruses were designed by Genomeditech (China) and transfections were conducted according to the manufacturer’s instructions. Briefly, SKOV3 cells were seeded in 6-well plates and infected with lentivirus in the presence of 6 μg/ml polybrene. Blasticidin (20 μg/ml), neomycin (100 μg/ml) and hygromycin (200 μg/ml) were used for positive cell selection.

### Characterization of HPMCs

HPMCs were characterized by immunohistochemistry (IHC) using antibodies directed against vimentin (R&D System, MAB21052), cytokeratin (Novus Biologicals, NB100–2756), CD45 (R&D System, MAB1430) and factor VII (KenGEN BioTECH, KGZA0084). Five random fields were analyzed at ×200 magnification. HPMCs stain positive for the mesenchymal markers vimentin and cytokeratin, but negative for the macrophage marker CD45 and the endothelial marker factor VII. Hematoxylin and eosin (H&E) stained slides were used for cell morphology assessment. HPMCs were successfully isolated from 6 EOC patients and 6 benign ovarian tumor patients.

### Histological and immunohistochemical analysis of the ITGA5B1/AEP complex

AEP and integrins were detected trough incubation of tissue sections overnight at 4 °C with anti-legumain (Abcam, ab125286), anti-integrin α5 (Abcam, ab150361) and anti-integrin β1 (CST, 9699) antibodies. Next, the sections were incubated with HRP-conjugated anti-goat IgG (R&D Systems, HAF017) and anti-rabbit IgG (CST, 7074) for 60 min at 37 °C. After rinsing three times for 5 min in PBS, the sections were incubated with DAB for 30 s, counterstained with hematoxylin and dehydrated. Evaluation was performed on five fields at ×200 magnification for AEP and integrin expression by two independent researchers. The AEP staining percentage (PP, percentage of positive cells) was scored as 1, 1%–25%, 2, 26%–50%, 3, 51%–75% or 4, 76%–100%. The AEP staining intensity (SI) was scored as 0 (negative), 1 (weak), 2 (moderate) or 3 (strong). Immunoreactive scores (IRS) were obtained by combining the PP and SI scores. The IRS were divided into the following groups: 0–3 (negative), 4–6 (weak positive), 7–9 (positive) and 10–12 (strong positive) [31].

### Immunofluorescence confocal microscopy analysis of the ITGA5B1/AEP complex

Detection of integrin α5, integrin β1 and AEP in HPMCs was performed using anti-integrin α5 (Abcam, ab150361), anti-integrin β1 (Abcam, ab134179) and anti-legumain (R&D, AF2199) antibodies. Cell nuclei were counterstained with DAPI (Sigma, D9542). Alexa Fluor 488-conjugated anti-rabbit antibody (Jackson, 111–545-003) was used for the detection of anti-integrin antibodies, and Cy3-conjugated anti-goat antibody (Jackson, 705–165-003) was used for the detection of anti-legumain antibody. Images were taken using a Zeiss LSM 510 laser confocal microscope. Quantitative analyses were performed in 5 random fields by counting the number of cells at ×200 magnification.

### Co-immunoprecipitation and Western blotting

Cells in 10 cm dishes were prepared for co-IP according to the manual of the Pierce™ Co-Immunoprecipitation Kit (ThermoFisher, 26,149). After overnight incubation with anti-legumain antibody (R&D, AF2199) the protein samples were analyzed by Western blotting (WB). Total proteins were obtained after cell lyses with RIPA and separated by 10% SDS PAGE. Next, the proteins were transferred to polyvinylidene fluoride membranes (Millipore, IPVH00010). After blocking with 5% non-fat milk for 2 h, the membranes were incubated with primary antibodies at 4 °C overnight and subsequently incubated with a secondary HRP-conjugated antibody for 1 h at room temperature. The signals were visualized using a chemiluminescent HRP substrate (Millipore, WBKLS0500). FAK, phospho-FAK, Akt and Erk were evaluated as downstream members of the HPMC proliferation and migration pathway. For the assessment of epithelial-mesenchymal transition (EMT) an EMT Antibody Sampler Kit was used (CST, 9782).

### Quantitative RT-PCR

RNA was collected using TRIzol, reverse-transcribed into cDNA and subjected to PCR using a PrimeScript RT-PCR kit (Takara, DRRo14A). The primer sequences used were as follows: Legumain forward primer 5`-GATGAACCACCTGCCGGATAA-3`, Legumain reverse primer 5`-CATCATAGTAACAGGCGTAGGACGA-3`. Integrin α5 forward primer 5`-AGACATTCGATCCCTCTACAACT-3` and reverse primer 5`-AATCGGCCAAACTCATCATGG-3`**.** Integrin forward primer β1 primer 5`-CAAGAGAGCTGAAGACTATCCCA-3` and reverse primer 5`-TGAAGTCCGAAGTAATCCTCCT-3`. ZO-1 forward primer 5`-CAACATACAGTGACGCTTCACA-3` and reverse primer 5`-CACTATTGACGTTTCCCCACTC-3`. E-cadherin forward primer 5`-CAACATACAGTGACGCTTCACA-3` and reverse primer 5`-CACTATTGACGTTTCCCCACTC-3`. Claudin-1 forward primer 5`-CCTCCTGGGAGTGATAGCAAT-3` and reverse primer 5`-GGCAACTAAAATAGCCAGACCT-3`. ZEB1 forward primer 5`-GATGATGAATGCGAGTCAGATGC-3` and reverse primer 5`-ACAGCAGTGTCTTGTTGTTGT-3`. N-cadherin forward primer 5`-TCAGGCGTCTGTAGAGGCTT-3` and reverse primer 5`-ATGCACATCCTTCGATAAGACTG-3`. β-catenin forward primer 5`-CCTATGCAGGGGTGGTCAAC-3` and reverse primer 5`-CGACCTGGAAAACGCCATCA-3`. Snail forward primer 5`-TCGGAAGCCTAACTACAGCGA-3` and reverse primer 5`-AGATGAGCATTGGCAGCGAG-3`. Slug forward primer 5`-CGAACTGGACACACATACAGTG-3` and reverse primer 5`-CTGAGGATCTCTGGTTGTGGT-3`. GAPDH forward primer 5`-GGAGCGAGATCCCTCCAAAAT-3` and reverse primer 5`-GGCTGTTGTCATACTTCTCATGG −3`. The amplification conditions for 40 cycles consisted of denaturation at 95 °C for 10 s and annealing at 60 °C for 32 s. The fold change was calculated as 2-ΔΔCt, where ΔΔCt = ΔCt treatment-ΔCt control and ΔCt = Ct target gene-Ct GAPDH.

### Exosome isolation and detection

Sera and ascites from EOC and benign ovarian tumor patients were centrifuged for 30 min at 2500 rpm to remove cell debris, followed by incubation with a Total Exosome Isolation Kit (SBI, EXOQ5A-1) overnight according to the manufacturer’s instructions and centrifugation at 10000 xg for 1 h. To isolate exosomes from SKOV3, the cells were cultured in serum-free RPMI-1640 medium for 24 h. Next, the supernatants were collected and centrifuged two times (1000 xg for 10 min and 3000 xg for 30 min to remove cells and/or fragments, followed by the addition of Total Exosome Isolation Reagent (Life Technologies, 4,478,359) overnight and centrifugation at 10,000 xg for 1 h at 4 °C. The resulting exosomes were resuspended in PBS and stored at −80 °C. The concentration of exosomes was determined using a BCA Protein Assay.

To detect exosomes derived from SKOV3 cells acting on HPMCs, they were labeled with SYTO ® RNA Select™ Green Fluorescent Cell Stain (Life Technology, S-32703). RNA was detected by bright green fluorescence (~490/530 nm) and the membranes were labeled with a red fluorescent cell stain (~589/617 nm) for 20 min at 37 °C. Next, the medium was removed and the HPMCs were washed three times with PBS for confocal microscopy.

### Exosome authentication

Three methods were used to identify exosomes, i.e., electron microscopy, Nanoparticle Tracking Analysis (NTA) and Western blotting. For electron microscopy, exosome pellets (see above) were re-suspended in PBS and dropped onto a carbon-coated copper electron microscope grid as previously described [18]. The exosomes were evaluated under a Tecnai G2 F20 ST transmission electron microscope. NTA was performed using ZetaView re-suspended in 500 μl PBS. Exosome biomarkers were detected by Western blotting using antibodies directed against the tetraspanin molecule CD63 (Abcam, ab216130), CD9 (Abcam, ab92726), CD81 (Abcam, ab109201) and Tsg101 (Abcam, ab125011).

### Soluble protein analysis

AEP, ITGA5 and ITGB1 levels were detected in sera and ascites samplers by ELISA using a Human Total Legumain DuoSet ELISA kit (R&D Systems, DY4769), a Human Integrin α5 ELISA kit (R&D Systems, DY1864–05) and a Human Integrin β1 Antibody kit (R&D Systems, MAB17784).

### Co-culture system and CCK8 assay

For exosome and HPMC co-culture, SKOV3 exosomes were isolated as described above, and HPMCs were cultured in a 96-well plate (1500 cells/well). Exosomes were added daily to the HPMCs at 50 μg/ml culture medium. At 24, 48, 72, 96 and 120 h, 10 μl CCK8 solution (Beyotime, C0041) was added to each well and incubated for 2 h at 37 °C. Absorption at 450 nm was measured using a microplate reader (Thermo Labsystems, Finland).

### Invasion assay

Cell invasion was evaluated using a Transwell chamber (8 μm pore; Corning, 3422). To this end, cell suspensions (6 × 10^4^ cells) in serum-free DMEM were added to the upper chamber with Matrigel (BD Biosciences, 356,234). DMEM containing 10% FBS was added to the bottom chamber. After 24 h, the cells on the upper surface were removed and the cells that migrated to the lower surface were stained with 0.2% crystal violet for 10 min. The cells were counted under a microscope (Olympus) using five random fields.

### In vivo experiment

Female athymic nude mice (42; 6 weeks of age, 15–17 g) were purchased from Shanghai Slac Laboratory Animal Center and bred under SPF conditions in Tongji University School, Shanghai, China. The mice were randomly divided into 7 groups, and subjected to orthotopic injection with 10 μl PBS containing 5 × 10^5^ SKOV3-luc cells. Five of them were matched with the in vitro experiments, i.e., the SKOV3-WT group, the SKOV3-NC (for OE) group, the SKOV3-ITGASB1/AEP-OE group, the SKOV3-NC (for KD) group and the SKOV3-ITGASB1/AEP-KD group. The other two groups were based on the SKOV3-ITGASB1/AEP-OE group, and AEP inhibitor [35] or dimethylamiloride (DMA) [36] were added every week. The tumors in the mice were examined for luciferin expression using D-luciferin (100 mg/kg, Invitrogen, Life Technologies, USA), and images were captured using a Night OWL IILB 983 instrument to assess tumor development every week. Image analyses were carried out using IndiGo software. All animal experiments were approved by the Medical Animal Care of Tongji University, and the animals were cared for according to the recommended use of laboratory animals.

### Statistics

Data analyses were performed using SPSS version 20.0 statistical software (SPSS Inc., Chicago, IL). The results are presented as mean ± SEM. All experiments were repeated at least trice. The non-parametric test, the LSD test, Fisher’s exact test and Levene’s test (two-tailed) were used to determine *p* values. Overall survival (OS) was calculated using the Kaplan-Meier method. Continuous variables in the figures are presented as mean ± SEM, and in the figures * denotes a *p* value < 0.05, ** denotes a *p* value < 0.01, *** denotes a *p* value < 0.001 and ^#^ denotes a *p* value > 0.05. A *p* value < 0.05 was considered statistically significant.

### Study approval

The human studies and animal protocols were approved by the Institutional Review Board of Shanghai First Maternity and Infant Hospital. Written informed consent was obtained from all study participants or their guardians.

## Results

### ITGA5B1 and AEP are highly expressed in EOC-HPMCs and co-localize in EOC cells and EOC-HPMCs

**Fig. 1:**
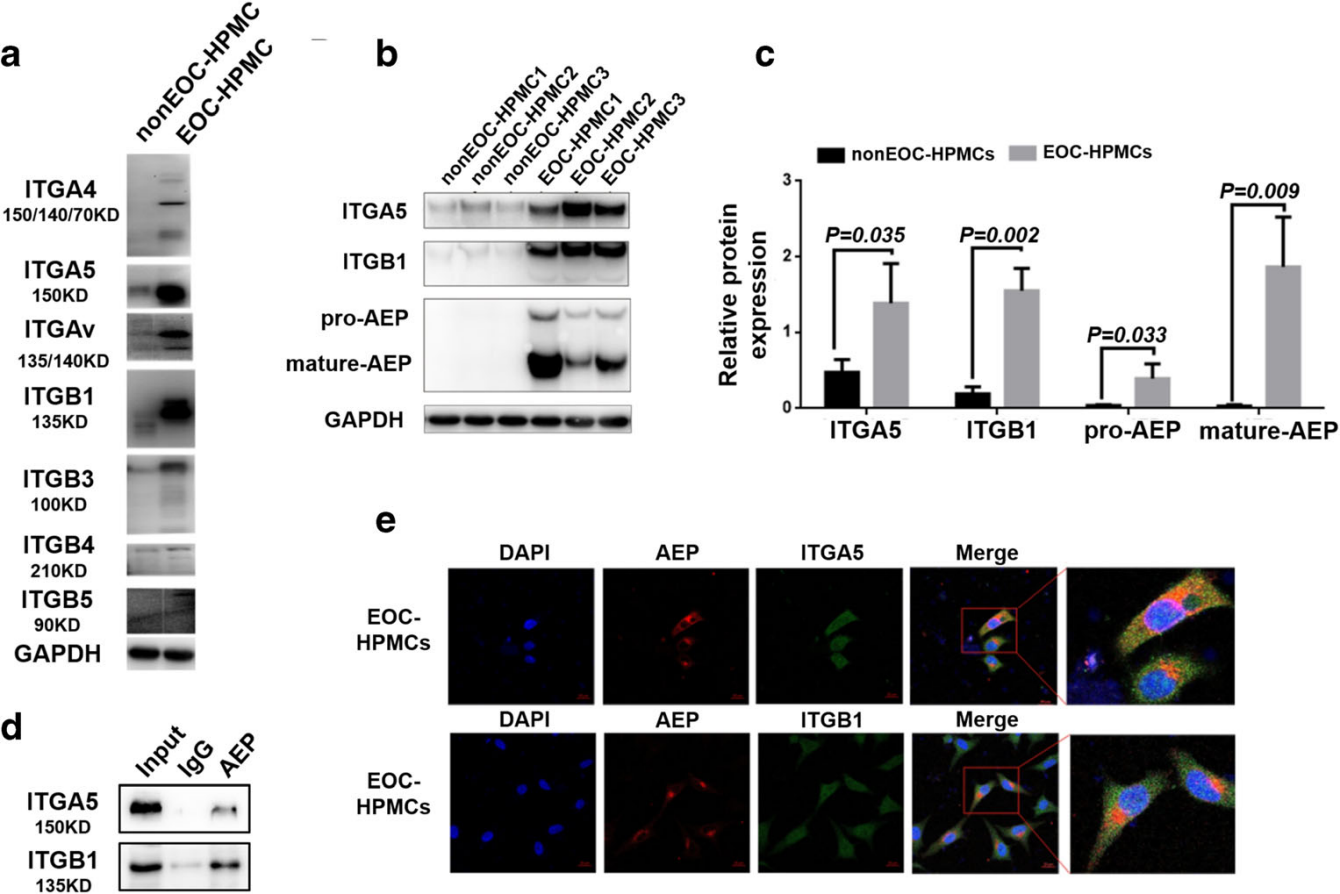
ITGA5B1 and AEP are highly expressed in EOC-HPMCs and co-localize in EOCs and EOC-HPMCs. **a** Western blot analysis of integrin subtypes (ITGA4, ITGA5, ITGAv, ITGB1, ITGB3, ITGB4 and ITGB5) in non EOC-HPMCs and EOC-HPMCs. **b** Western blot analysis of ITGA5, ITGB1, pro-AEP and AEP proteins in non EOC-HPMCs and EOC-HPMCs. **c** Gray values of Western blots were measured using ImageJ software. **d** Co-IP verifying the interaction of AEP with ITGA5B1. **e** Confocal microscopy images of AEP and ITGA5B1 co-localization in EOC-HPMCs. Scale bars, 20 μm

HPMCs from EOC patients (EOC-HPMCs) were identified using immunohistochemistry and HE staining (Fig. S1). Integrin subtypes, including integrin α4 (ITGA4), integrin α5 (ITGA5), integrin αv (ITGAv), integrin β1 (ITGB1), integrin β3 (ITGB3), integrin β4 (ITGB4) and integrin β5 (ITGB5), were assessed by Western blotting to compare EOC-HPMC and non EOC-HPMC subtypes (Fig. 1a). The integrin subtypes ITGA5 and ITGB1 showed the largest differences. In addition, we found that ITGA5B1 and AEP were expressed at higher levels in EOC-HPMCs than in non EOC-HPMCs (Fig. 1b). We calculated the gray values of each integrin subtype using GAPDH as an internal control (Fig. 1c). ITGA5B1 was found to be most abundant in all subtypes. In addition, we performed co-IP experiments revealing interaction of AEP with ITGA5B1 (Fig. 1d). HPMCs derived from 3 EOC patients were evaluated by immunofluorescence whereby fluorescence signals in different channels were superimposed. We found that ITGA5B1 was expressed in EOC-HPMCs (Fig. 1e) and EOC-derived SKOV3 cells (Fig. S2a), and that ITGA5B1 and AEP co-localized in the cytoplasm.

### ITGA5B1 complexes with AEP in EOC-HPMCs and is overexpressed in exosomes from sera and ascites from EOC patients

**Fig. 2:**
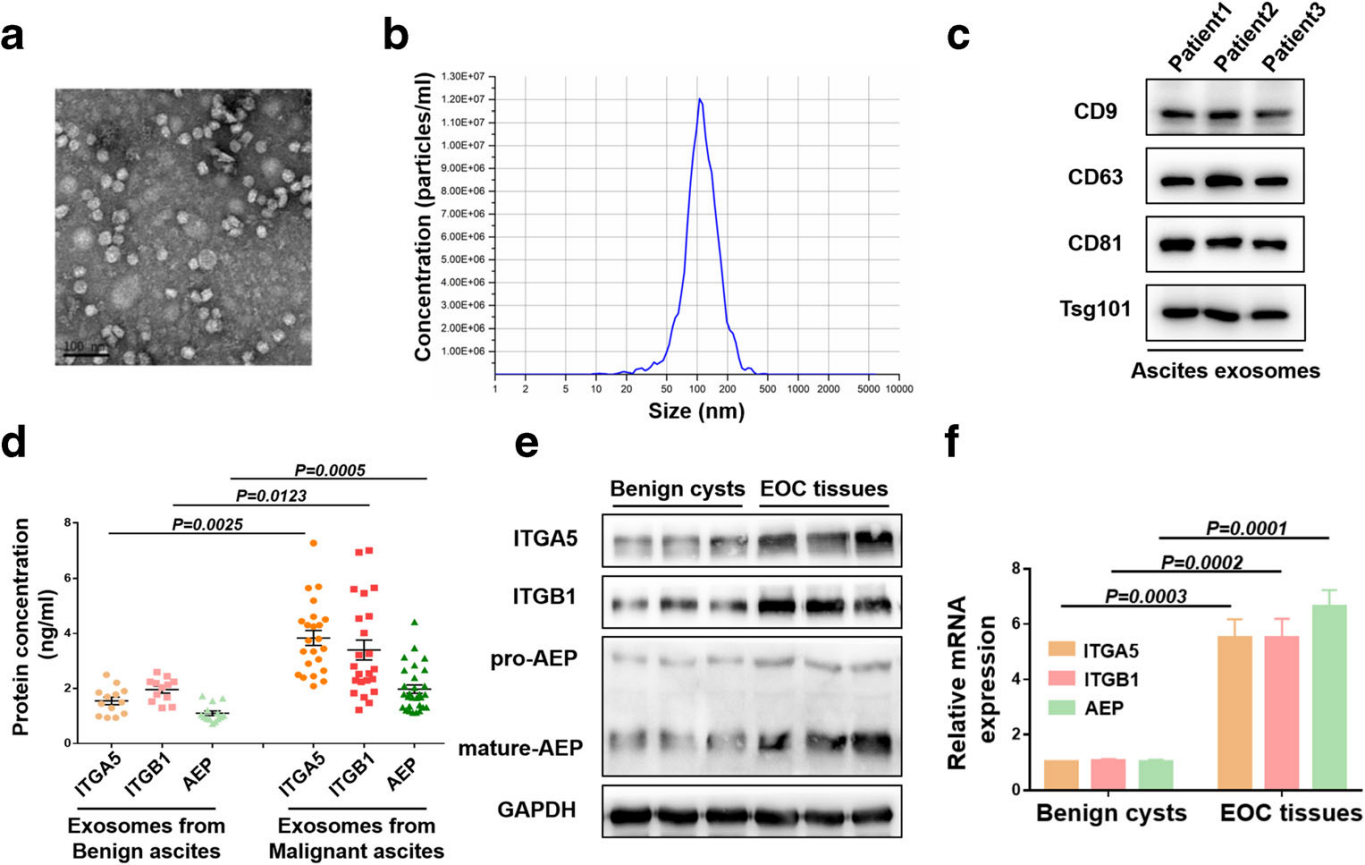
ITGA5B1/AEP is overexpressed in serum and ascites exosomes from patients with EOC. **a** Representative transmission electron microscopy (TEM) micrograph of exosomes derived from ascites. Scale bar, 100 nm. **b** NTA showing that the sizes of the exosomes range from 30 nm to 150 nm. **c** Western blot analysis of Tsg101, CD9, CD63 and CD81 in exosomes derived from ascites. **d** Expression of AEP, ITGA5 and ITGB1 in exosomes derived from ascites of EOC patients and non-EOC patients analyzed by ELISA. **e** and **f** Western blot and quantitative RT-PCR analyses of ITGA5, ITGB1 and AEP expression in benign ovarian cysts and EOC tissues

We collected sera and ascites from benign ovarian tumor patients, hepatocirrhosis patients and EOC patients, and purified exosomes from them. The exosomes were identified by electron microscopy (Fig. 2a), and NTA (Fig. 2b) and the biomarkers Tsg101, CD9, CD63 and CD81 were detected by Western blotting (Fig. 2c). We found that the expression of AEP was higher in ascites exosomes derived from EOC patients (1.735 ± 0.112 ng/ml) than in those derived from benign ovarian tumor patients or hepatocirrhosis patients (0.847 ± 0.051 ng/ml, *p* = 0.0005). The exosome integrins ITGA5 (3.835 ± 0.272 ng/ml to 1.548 ± 0.139 ng/ml, *p* = 0.0025) and ITGB1 (3.401 ± 0.363 ng/ml to 1.958 ± 0.125 ng/ml, *p* = 0.0123) in ascites showed the same trend as AEP (Fig. 2d). The expression of ITGA5, ITGB1 and AEP was also higher in serum exosomes derived from EOC patients than in those derived from benign ovarian tumor patients (Fig. S2b). In addition, we found that total ITGA5, ITGB1 and AEP levels in ascites from EOC patients showed no significant differences from those in ascites from benign ovarian tumor patients or hepatocirrhosis patients (Fig. S2c). These results indicate that exosomes show enrichment effects. We additionally collected 3 EOC tissues and 3 benign ovarian cysts, and analyzed protein and mRNA expression levels. We found that, compared to benign ovarian cysts, EOC tissues showed higher ITGA5, ITGB1 and AEP levels (Fig. 2e and f), which may be the source of exosomal ITGA5B1/AEP in malignant ascites and malignant serum.

### The exosomal ITGA5B1/AEP complex secreted by EOC cells affects the proliferation and migration of HPMCs

**Fig. 3:**
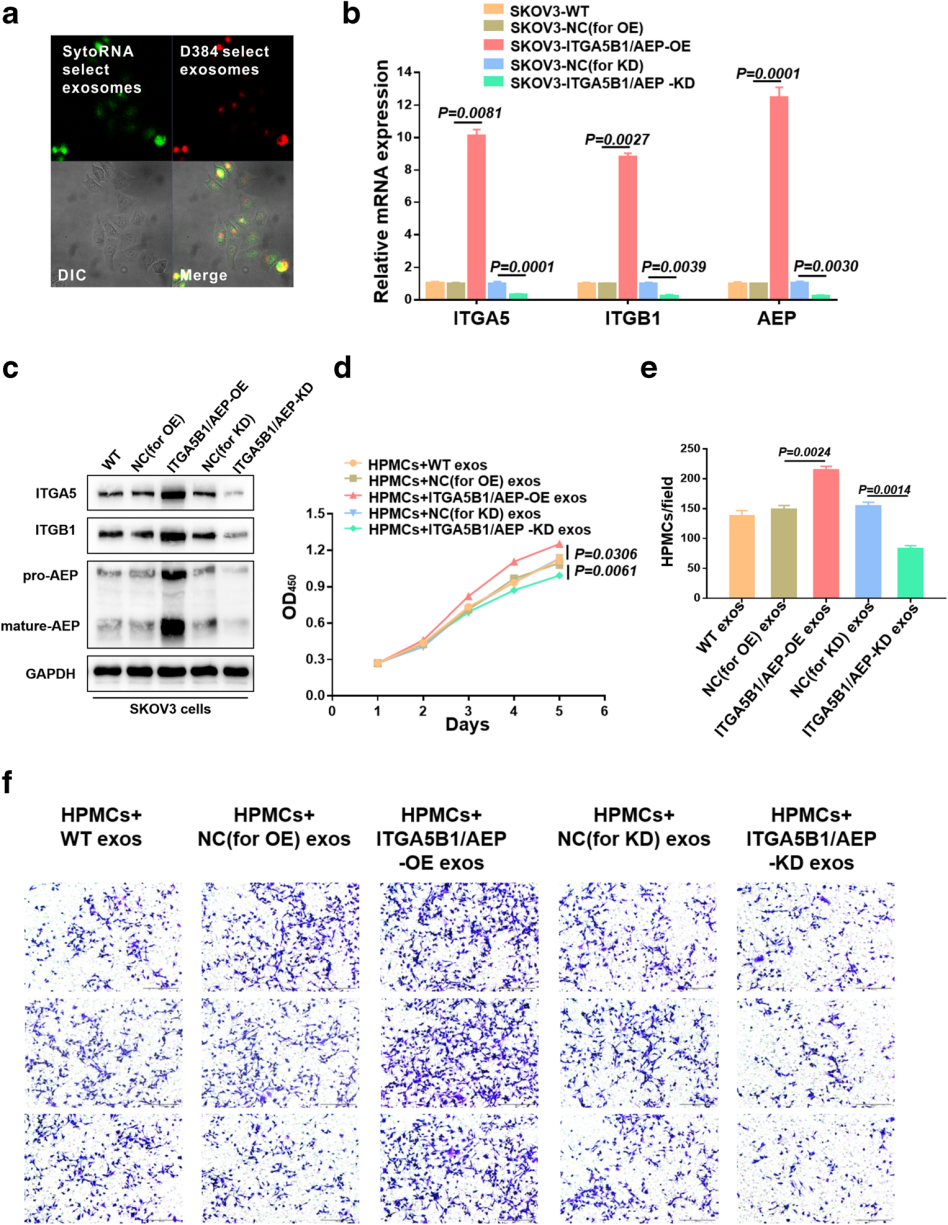
The Exosomal ITGA5B1/AEP complex secreted by EOC cells plays an important role in the proliferation and migration of HPMCs in vitro. **a** Unlabeled HPMCs were incubated with SKOV3-derived exosomes labeled with SytoRNA and D384. The DIC (differential interference contrast) presents the morphology of HPMCs that were not fluorescent. The data depict a typical image of 3 independent experiments. **b** and **c** Transfection efficiency of ITGA5, ITGB1 and AEP was detected by RT-PCR and Western blotting after establishing stable ITGA5B1/AEP-OE and ITGA5B1/AEP-KD SKOV3 cell lines. The data depict a typical image of 3 independent experiments. **d** and **e** Proliferation and migration capacities of HPMCs after incubation with exosomes derived from WT, NC (for OE), ITGA5B1/AEP-OE, NC (for KD) and ITGA5B1/AEP-KD cells using CCK8 and Transwell assays, respectively. **f** Crystal violet staining showing the migration of five groups. Typical images of three independent experiments are shown. Scale bar, 200 μm

We used immunofluorescence to verify whether exosomes are taken up by HPMCs. RNAs (green) and membranes (red) of exosomes in HPMCs were evaluated using confocal microscopy (Fig. 3a). Next, we used lentiviral vectors to establish stable ITGASB1/AEP-overexpressing (OE) and ITGA5B1/AEP-knockdown (KD) SKOV3 cell lines. The expression level of ITGA5B1/AEP was detected by quantitative RT-PCR (Fig. 3b) and Western blotting (Fig. 3c). To determine whether the exosomal ITGA5B1/AEP complex is crucial for HPMC proliferation and migration, we collected exosomes from SKOV3-WT, SKOV3-NC (for OE), SKOV3-ITGA5B1/AEP-OE, SKOV3-NC (for KD) and SKOV3-ITGA5B1/AEP-KD cells. These exosomes were subsequently co-cultured with HPMCs. The results showed that the proliferation rate of HPMCs treated with exosomes from ITGA5B1/AEP-KD cells was lower than that of HPMCs treated with exosomes from NC (for KD) cells (*p* = 0.0061, Fig. 3d). In contrast, we found that HPMCs treated with exosomes from ITGA5B1/AEP-OE cells grew faster than those treated with exosomes from NC (for OE) cells (*p* = 0.0306, Fig. 3d). Similarly, the migration capacity of HPMCs treated with exosomes from ITGA5B1/AEP-KD cells was found to be lower than that of HPMCs treated with exosomes from NC (for KD) cells (*p* = 0.0014, Fig. 3e). The opposite tendency was noted in ITGA5B1/AEP-OE exosomes and NC (for OE) exosomes (*p* = 0.0024, Fig. 3e). Crystal violet staining depicted the migration abilities of the five groups (Fig. 3f). Taken together, these data indicate that the exosomal ITGA5B1/AEP complex secreted by EOC cells affects the proliferation and migration of HPMCs in vitro.

### HPMCs undergo ITGA5B1/AEP downstream FAK/Akt/Erk pathway changes and EMT after co-culture with EOC-derived exosomes

**Fig. 4:**
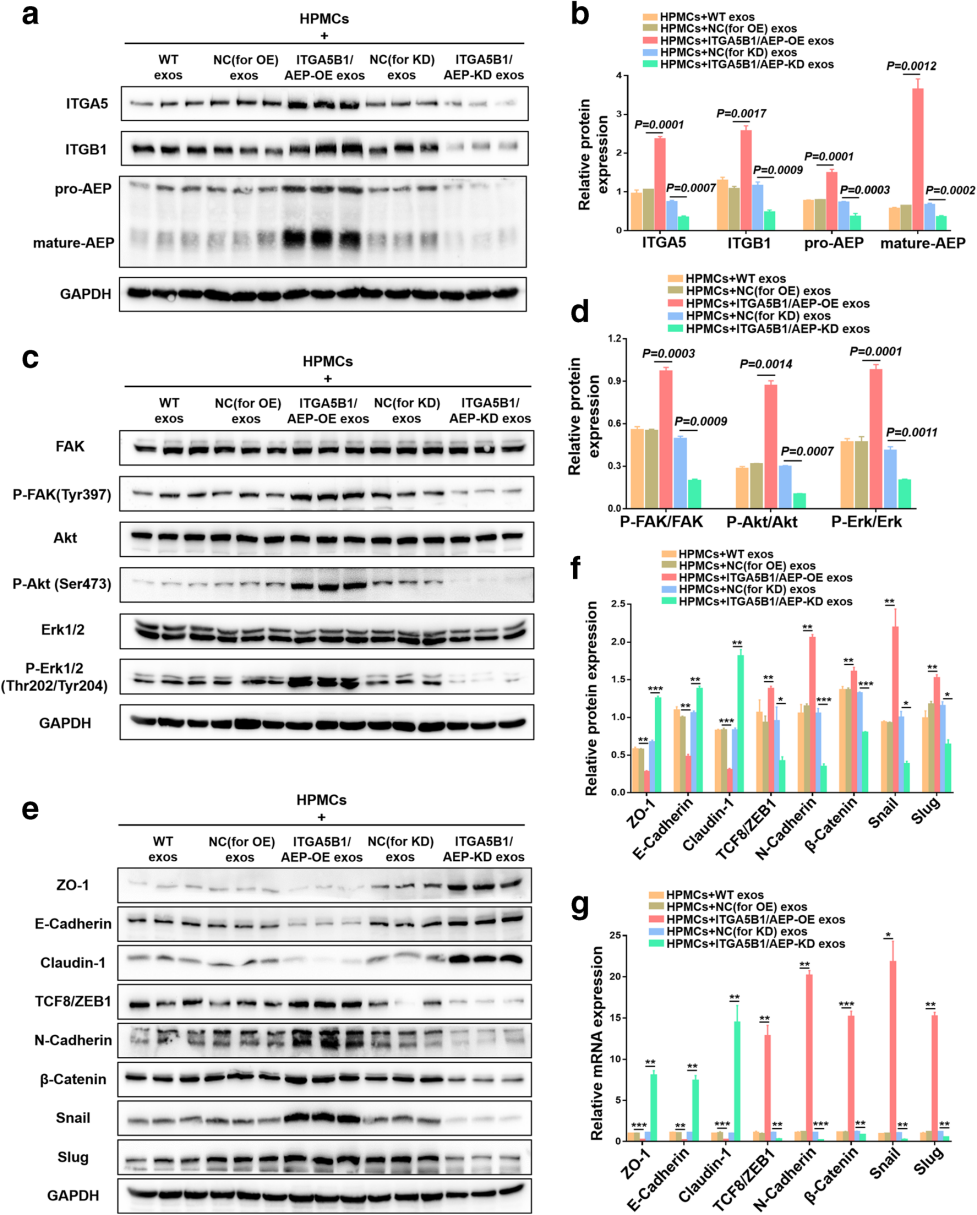
HPMCs undergo downstream FAK/Akt/Erk pathway changes and EMT after co-culture with EOC exosomes. **a** and **b** Western blot and gray value analyses showing ITGA5, ITGB1 and AEP expression in HPMCs after co-culture with WT exosomes, NC (for OE) exosomes, ITGA5B1/AEP-OE exosomes, NC (for KD) exosomes and ITGA5B1/AEP-KD exosomes. **c** and **d** Western blot and gray value analyses showing FAK, P-FAK, Akt, P-Akt, Erk and P-Erk expression in the five different groups. **e** and **f** Western blot and gray value analyses showing the EMT markers ZO-1, E-cadherin, claudin-1, ZEB1, N-cadherin, β-catenin, Snail and Slug. **g** RT-PCR analysis showing mRNA levels of EMT markers. All data presented are representative of three independent experiments

To assess changes in downstream pathways after exosomal ITGA5B1/AEP transfer to HPMCs, we collected HPMCs co-cultured with five different EOC-derived exosomes, namely WT exosomes, NC (for OE) exosomes, ITGA5B1/AEP-OE exosomes, NC (for KD) exosomes and ITGA5B1/AEP-KD exosomes. Using Western blotting we found that the HPMCs exhibited higher ITGA5, ITGB1 and AEP expression levels after co-culture with ITGA5B1/AEP-OE exosomes, and that those after co-culture with ITGA5B1/AEP-KD exosomes showed the opposite effect (Fig. 4a). The gray values of each blot were used to reflect the relative content (Fig. 4b). Next, we found that the proliferation and migration-related FAK/Akt/Erk pathways [37, 38] showed no changes in total FAK, Akt and Erk levels, but that the phosphorylation levels of each protein in the ITGA5B1/AEP-OE exosomes group increased, whereas they decreased in the ITGA5B1/AEP-KD exosomes group (Fig. 4c and d). In addition, we found that the levels of proteins involved in EMT, and the epithelial markers ZO-1, E-cadherin and claudin-1, were decreased in the ITGA5B1/AEP-KD exosomes group, but increased in the ITGA5B1/AEP-OE exosomes group. At the same time, we found that the mesenchyme-related proteins ZEB1, N-cadherin, β-catenin, Snail and Slug showed the opposite trend (Fig. 4e and f). Finally, quantitative RT-PCR revealed that the respective mRNA levels showed the same trend as the protein levels (Fig. 4g).

### Inhibiting exosome secretion or ITGA5B1/AEP complex function decreases peritoneal metastasis and overall survival in vivo

**Fig. 5:**
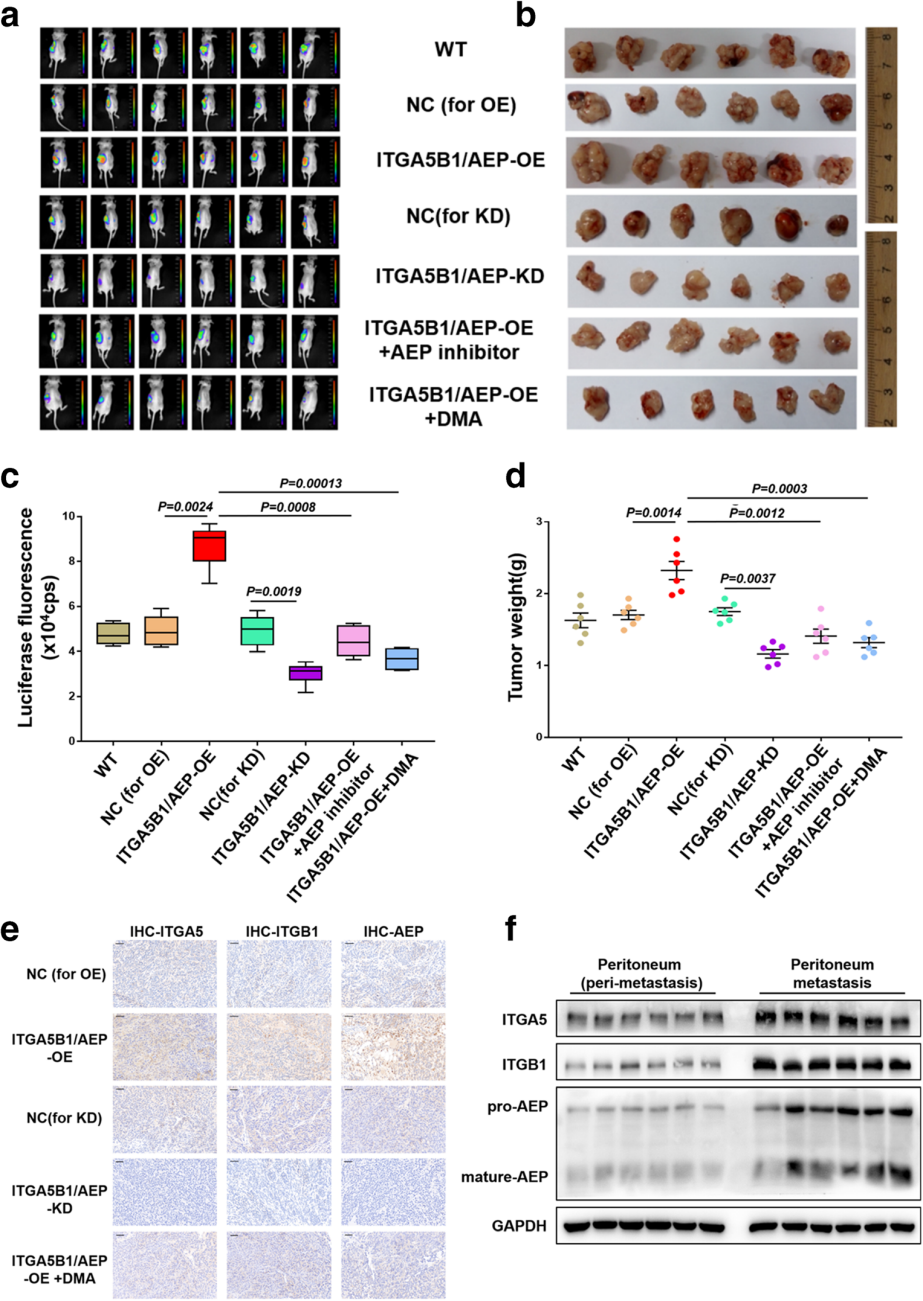
Inhibition of exosomal secretion or ITGA5B1/AEP function decreases peritoneal metastasis and overall survival in vivo. **a** Typical images of tumor flux (cps) at week 6 are presented. **b** After euthanasia, the tumor tissues were removed and presented. **c** The tumor flux of different groups recorded by bioluminescence imaging is presented. **d** Tumor weights of different groups. **e** Immunohistochemical staining performed on peritoneal metastases in mice of different groups. Scale bar, 20 μm. **f** Western blot analysis showing the expression of ITGA5, ITGB1 and AEP in peritoneal metastasis and corresponding peri-metastasis tissues

To investigate the role of the ITGA5B1/AEP complex in EOC peritoneal metastasis in vivo, different groups were denoted according to the different dispositions, i.e., SKOV3-WT group, SKOV3-NC (for OE) group, SKOV3-ITGA5B1/AEP-OE group, SKOV3-NC (for KD) group, SKOV3-ITGA5B1/AEP-KD group, SKOV3-ITGA5B1/AEP-OE + AEP inhibitor group and SKOV3-ITGA5B1/AEP-OE + DMA group. After orthotopic injecting different SKOV3-luc cells in nude mice, we used a bioluminescence imaging system to assess tumor growth. The tumor flux (cps) was recorded every week, and typical images at week 6 are presented (Fig. 5a). The ITGA5B1/AEP-OE group (8.732 ± 0.384 × 10^4^ cps) led to an enhancement in total tumor flux compared to that of the NC (for OE) group (4.917 ± 0.263 × 10^4^ cps), the ITGA5B1/AEP-OE + AEP inhibitor group (4.440 ± 0.259 × 10^4^ cps) or the ITGA5B1/AEP-OE + DMA group (3.670 ± 0.195 × 10^4^ cps). The ITGA5B1/AEP-KD group (3.032 ± 0.189 × 10^4^ cps) showed reduced tumor growth (Fig. 5c). These results were in conformity with the tumor weight (Fig. 4d) and tumor volume (Supplemental Table S1) results.

After euthanasia, the tumor tissues were removed (Fig. 5b) and found to be accompanied by metastases to the peritoneum, spleen, liver, stomach, pancreas, lymph nodes and ascites (Table 1). More tumor metastases were observed in the ITGA5B1/AEP-OE group than in the other six groups. Immunohistochemical staining was performed on peritoneal metastases in the different groups (Fig. 5e). The peritoneal metastases and the corresponding peri-metastasis tissues were collected for Western blot analysis. We found that the expression of ITGA5, ITGB1 and AEP in the peritoneal metastases was significantly higher (Fig. 5f). Together, these data indicate that the exosome ITGA5B1/AEP complex promotes peritoneal metastasis of EOC in vivo, and that inhibition of exosome secretion or ITGA5B1/AEP complex function may decrease EOC peritoneal metastasis.

**Table 1:** Number of tumor metastases in an orthotopic model of ovarian cancer (*n* = 6)

Group	Peritoneal metastases	Spleen	Liver	Stomach	Pancreas	Lymph nodes	Ascites
SKOV3-WT	2/6	2/6	1/6	2/6	1/6	1/6	2/6
SKOV3-NC (for OE)	1/6	3/6	2/6	1/6	1/6	1/6	1/6
SKOV3-ITGA5B1/AEP-OE	4/6	5/6	3/6	3/6	3/6	2/6	3/6
SKOV3-NC (for KD)	2/6	1/6	3/6	2/6	1/6	1/6	1/6
SKOV3-ITGA5B1/AEP-KD	2/6	1/6	1/6	1/6	1/6	1/6	1/6
SKOV3-ITGA5B1/AEP-OE+AEP inhibitor	2/6	2/6	0/6	1/6	1/6	0/6	0/6
SKOV3-ITGA5B1/AEP-OE+DMA	1/6	1/6	0/6	0/6	0/6	0/6	1/6

### Expression of the ITGA5B1/AEP complex in clinical specimens is associated with EOC progression and metastasis

**Fig. 6:**
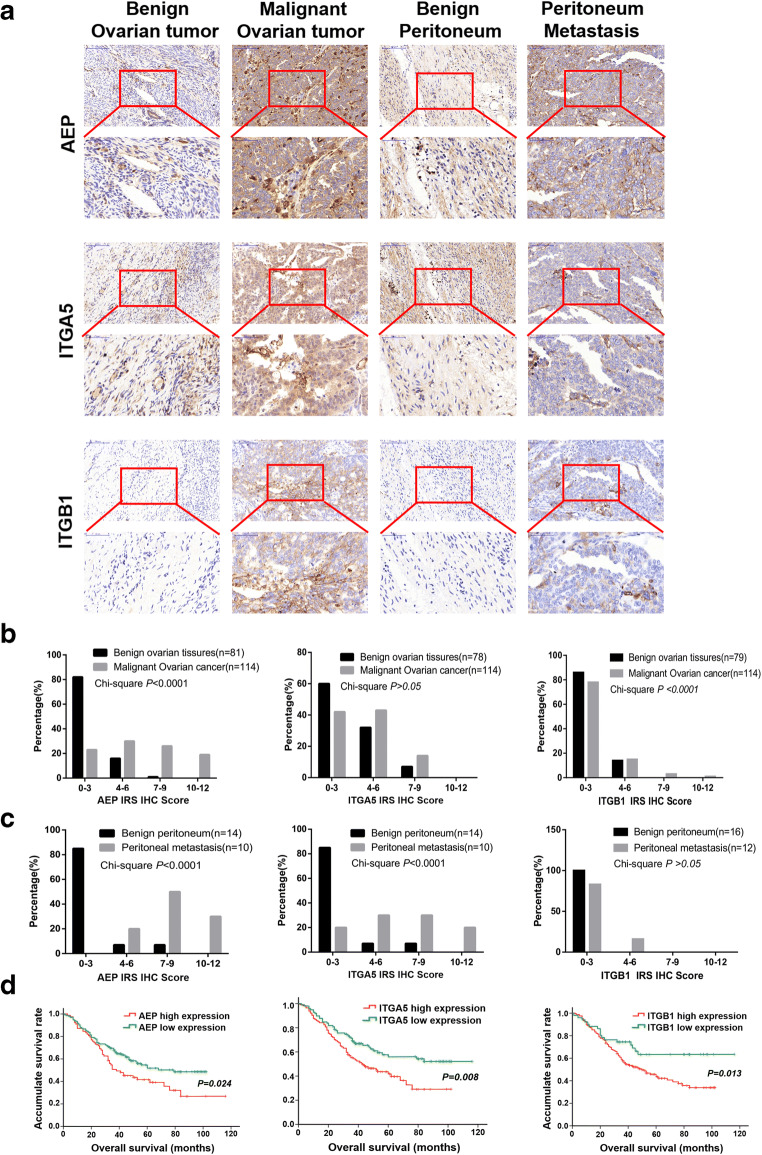
High expression of ITGA5B1/AEP in EOC primary tissues and metastatic tissues is negatively associated with overall survival. **a** Representative immunohistochemistry (IHC) staining of the AEP protein in EOC tissues (*n* = 153), benign ovarian tumor tissues (*n* = 81), benign peritoneum tissues (*n* = 14) and peritoneal metastasis tissues (*n* = 15). **b** and **c** An IRS scoring system was used to evaluate the expressions of AEP, ITGA5 and ITGB1. **d** EOC patients with a high ITGA5B1/AEP expression exhibited a significantly lower overall survival

To assess the expression pattern of the ITGA5B1/AEP complex, 81 benign ovarian tumor tissues, 14 benign peritoneal tissues, 114 EOC tissues and 10 peritoneal metastatic tissues were evaluated by immunohistochemistry. To evaluate ITGA5B1/AEP complex expression, we used the IRS scoring system (IRS = Staining Intensity (SI) × Percentage of Positive cells (PP)) [31]. In doing so, we found that the expression of AEP was higher in EOC and peritoneal metastatic tissues than in benign ovarian tumor and benign peritoneal tissues (Fig. 6a–c). Similar results were obtained for ITGA5 and ITGB1 (Fig. 6a–c). Together, these data suggest that a high expression of the ITGA5B1/AEP complex may be associated with EOC progression and metastasis. Clinical characteristics of the EOC patients and univariate analyses of overall survival rates are listed in Table 2. EOC patients with a high expression of the ITGA5B1/AEP complex exhibited a significantly lower overall survival rate than patients with a low expression of the ITGA5B1/AEP complex (Fig. 6d).

**Table 2:** Univariate analyses of OS in 153 EOC patients

Variables	N	Overall survival (OS) univariate analysis (Mean ± SEM)	*p* value
*Age*			
< 50	67	46.45 ± 2.92	0.153
≥ 50	86	45.71 ± 2.43	
*Clinical stage*			
I + II	38	65.04 ± 4.47	<0.001
III + IV	115	39.51 ± 1.85	
*Histological subtype*			
SOC	131	42.40 ± 1.95	0.005
NSOC	22	59.77 ± 5.36	
*Histological grade*			
Low	47	60.42 ± 4.11	0.001
High	106	39.71 ± 1.93	

## Discussion

Integrins are known to regulate cell-cell and cell-matrix interactions and it has amply been reported that integrins may be involved in tumor progression and metastasis [39–41]. According to the “seed-and-soil” hypothesis, metastatic organotropism is one of the characteristics of cancer [42]. Exosome proteomics revealed distinct integrin expression patterns, such as those of the integrins α6β4 and α6β1, which have been associated with lung metastasis, whereas that of integrin αvβ5 has been linked to liver metastasis [43]. Integrin functions may thus vary with its different subunits, and it is therefore important to consider integrin subtypes when exploring their effects in different cancers [16–18]. In this study, we isolated HPMCs from EOC patients and assessed integrin subtypes. We found that ITGA5B1was highly expressed in HPMCs from EOC patients. More importantly, we found using immunofluorescence confocal microscopy that ITGA5B1 co-localized with AEP in HPMCs from EOC patients.

AEP, also known as legumain, is synthesized as a zymogen-like endosomal protease, and AEP can undergo auto-proteolytic maturation under acidic pH conditions to catalytic activation. In addition, AEP has been found to be predominantly localized in late endosomes and lysosomes [44, 45]. More recently, AEP has been reported to regulate innate immune responses via its participation in the maturation of toll-like receptors (TLRs) [46]. Others found that AEP was highly expressed on the surface of tumor-associated macrophages (TAMs), and that these AEP^+^ TAMs play a critical role in promoting tumor development and angiogenesis. Targeting AEP on TAMs may, therefore, represent a novel anticancer strategy [47, 48]. AEP expression has also been found to be related to clinicopathologic and biological variables in colorectal cancer [49]. So far, however, the relationship between ITGA5B1and AEP expression in EOC has remained unknown. In 2003 it was reported that AEP may co-localized with integrins [50]. In the present study, we found that both high ITGA5B1 and AEP levels were found in exosomes derived from sera and ascites of EOC patients. The mechanism underlying exosomal ITGA5B1/AEP complex EOC peritoneal metastasis promotion warrants further discussion.

Exosomes are known to contain microRNAs, mRNAs and proteins, to be released from normal cells and cancer cells and to play important roles in cell-to-cell communication [51, 52]. To explore the functional role of the exosomal ITGA5B1/AEP complex, we established stable AEP-KD SKOV3 cell lines, and confirmed that suppression of the exosomal ITGA5B1/AEP complex reduced HPMC proliferation and migration. Conversely, we found that the exosomal ITGA5B1/AEP complex from AEP-OE SKOV3 cells could promote HPMC proliferation and migration. Finally, we found using primary clinical samples that the ITGA5B1/AEP complex was highly expressed in exosomes derived from sera and ascites of patients with EOC. Our results also indicated that tumor-derived exosomes taken up by organ-specific cells may prepare the pre-metastatic niche [43], which in turn may explain the mechanism underlying peritoneal EOC metastasis.

Our data provide new insights into how the exosomal ITGA5B1/AEP complex may promote HPMC proliferation and migration both in vivo and in vitro. We found that HPMCs underwent downstream FAK/Akt/Erk pathway changes and EMT after co-culture with EOC-derived exosomes. A putative HPMC feedback on cancer cells requires, however, further investigation. Data retrieved from clinical specimens indicated that ITGA5B1 and AEP were overexpressed in both primary EOC tissues and metastatic lesions, and that high ITGA5B1/AEP levels were associated with a poor prognosis. As such, the ITGA5B1/AEP complex may serve as a prognostic factor in EOC patients.

**Fig. 7:**
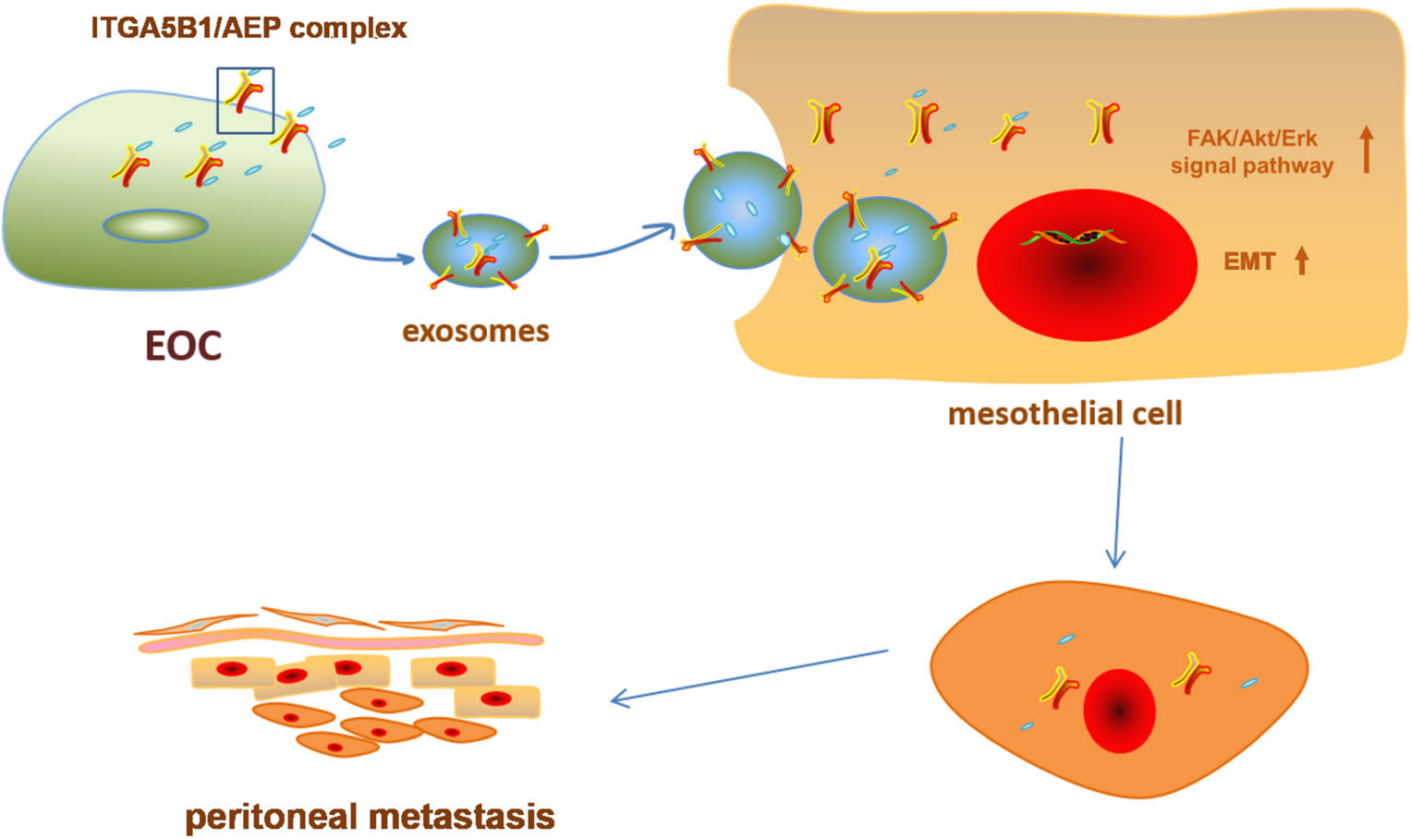
Diagram illustrating our conclusions. EOC cells transfer exosomes that are enriched in ITGA5B1/AEP to HPMCs, and these exosomes promote the proliferation and migration of HPMCs. In addition, HPMCs undergo phosphorylation of the FAK/Akt/Erk pathway as well as EMT, ultimately favoring EOC peritoneal metastasis

In conclusion, our data indicate that the exosomal ITGA5B1/AEP complex derived from EOCs may promote the proliferation and migration in HPMCs through regulating the FAK/Akt/Erk pathway as well as EMT, ultimately resulting in peritoneal metastasis (Fig. 7). These exosomes and the ITGA5B1/AEP complex may serve as prognostic factors and/or potential therapeutic targets for EOC.

## Electronic supplementary material

ESM 1 (DOCX 1506 kb)
