# Supplementary material for: The exosomal integrin α5β1/AEP complex derived from epithelial ovarian cancer cells promotes peritoneal metastasis through regulating mesothelial cell proliferation and migration
Source: Cell Oncol (Dordr). 2020 Feb 21;43(2):263–77. doi: 10.1007/s13402-019-00486-4 (PMC12990696; doi:10.1007/s13402-019-00486-4)
Supplement: Supplementary file 1 — (DOCX 1506 kb) [file 13402_2019_486_MOESM1_ESM.docx]

**Supplement data**


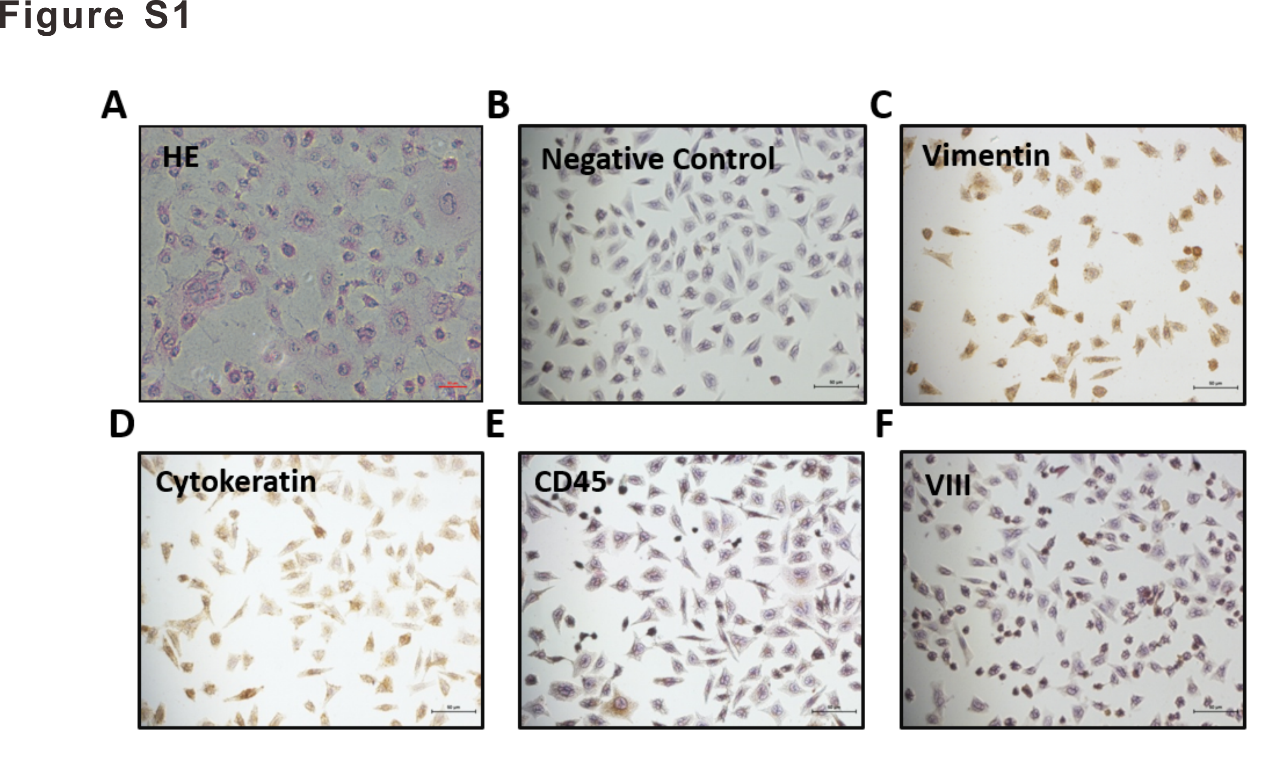


**Figure S1: Isolated and authentication of HPMCs from advanced EOCs.**

(A) Hematoxylin and Eosin stain of HPMCs. (B-E) Immunohistochemistry staining of HPMC with negative control, anti-vimentin, anti-cytokeratin, anti-CD45 and anti-VIII. x200, Scale bar, 100μm


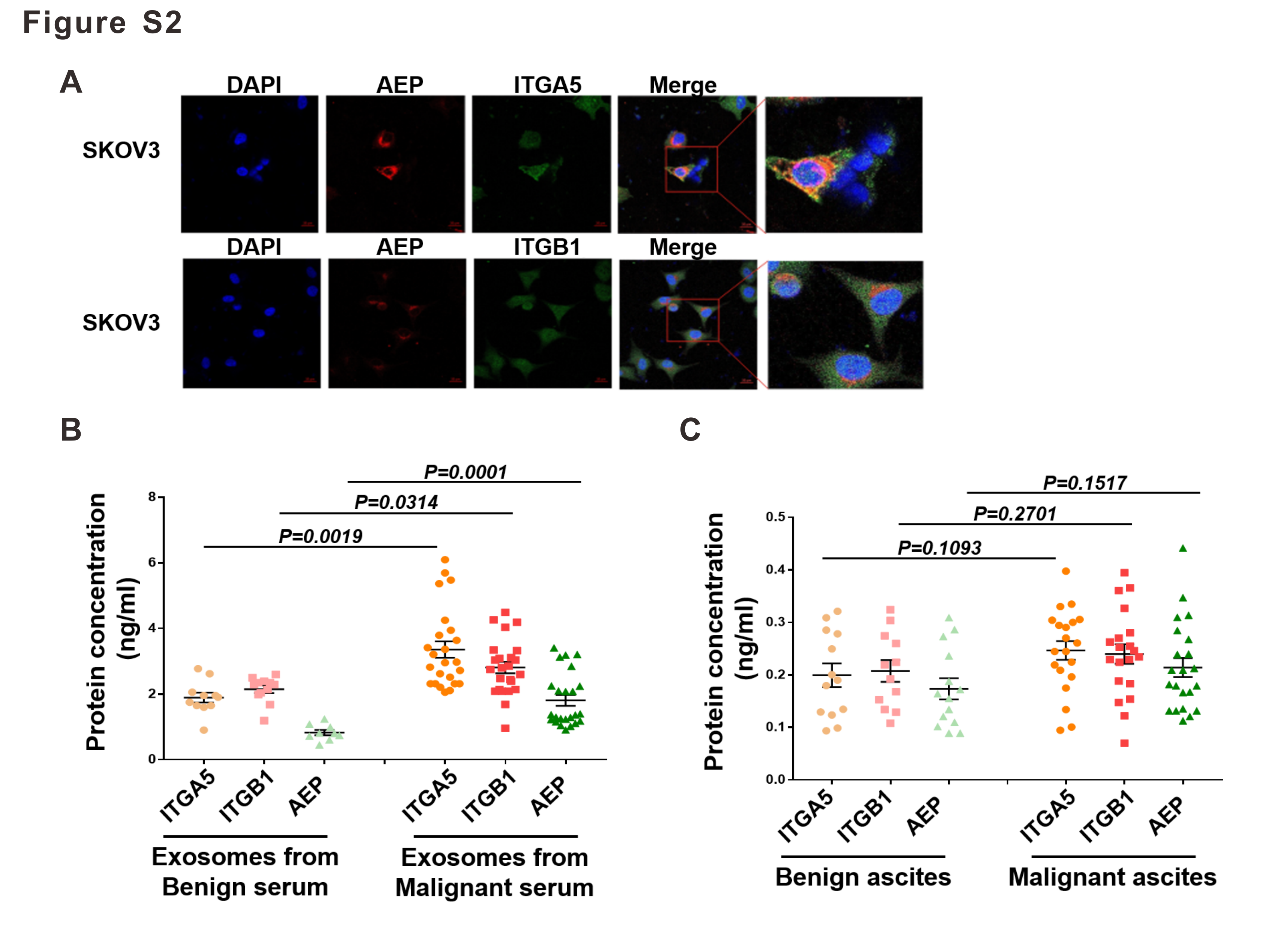


**Figure S2: ITGA5B1/AEP was overexpressed in circulating serum exosomes and ascites in EOC patients.** (A) Confocal microscopy image of colocalization of AEP and ITGA5B1 in SKOV3 cells, scale bars, 20 μm. (B) ELISA analyzed the expressions of AEP, ITGA5 and ITGB1 in exosomes from circulating serum in EOC patients and non-EOC patients. (C) ELISA analyzed the expressions of total AEP, ITGA5 and ITGB1 in ascites of EOC patients and non-EOC patients.

**Supplement Table 1. The tumor volume and weight in orthotopic model of ovarian cancer (Mean ± SEM, n=6). Non-parametric Test, Kruskal-Wallis Test.**

| **Group** | **Weight (g)** | **Volume(mm^3^)** |
| --- | --- | --- |
| **SKOV3-WT** | 1.627±0.102 | 2255.33±664.56 |
| **SKOV3-NC (for OE)** | 1.702±0.065 | 2460.50±766.17 |
| **SKOV3-ITGA5B1/AEP-OE** | 2.322±0.126 | 3866.50±1021.05 |
| **SKOV3-NC (for KD)** | 1.748±0.054 | 2320.25±461.72 |
| **SKOV3-ITGA5B1/AEP-KD** | 1.163±0.057 | 1188.17±327.40 |
| **SKOV3-ITGA5B1/AEP-OE+AEP inhibitor** | 1.407±0.099 | 1289.83±768.97 |
| **SKOV3-ITGA5B1/AEP-OE+DMA** | 1.317±0.070 | 797.33±155.02 |
